# A fast and effective solution for ingrown toenail: Creation of a 2-mm space between tissue and nail by sutureless lateral longitudinal excision

**DOI:** 10.1007/s00403-020-02177-9

**Published:** 2021-01-12

**Authors:** Ethem Unal, Sema Yuksekdag

**Affiliations:** 1 Department of General Surgery, Sancaktepe Martyr Prof. Dr. Ilhan Varank Training and Research Hospital, 34785 Istanbul, Turkey; 2 Department of General Surgery, Umraniye Training and Research Hospital, 34760 Istanbul, Turkey

**Keywords:** Ingrown toenail, Lateral excision, Longitudinal excision, Matrix excision, Matricectomy, Sutureless

## Abstract

Nails have both functional and aesthetic importance. Undertreatments cause frequent recurrences affecting its functionality, while over-treatment spoils the aesthetic view. To describe the most practical and aesthetic method to treat ingrown toenail. All patients with ingrown toenail who applied to outpatient clinics of General Surgery Department between 2013 and 2019 were enrolled. A 2-mm space between tissue and nail by lateral longitudinal excision was created with only minimal matricectomy, under local anaesthesia. A total of 2334 surgical procedures were performed in 2118 patients. Recurrence rate was 1.7% during 36-month follow-up, most (70.7%) in younger men (22 years). The location of the lesions (right/left, medial/lateral or bilateral) did not show difference (*p* > 0.05 for each). Predisposing factors were tight-fitting footwear (4.5%), incorrect nail-trimming (3%), genetic tendency (2.8%), obesity (2.1%) and trauma (0.75%); but each was *p* > 0.05. Mean operation time was 3 min. There was no important complication, except hematoma (0.89%) and infection (0.68%). Mean healing time was 10 days and patients returned to daily activities in 3 days. Longitudinal excision with minimal matricectomy technique provides all dead tissue and diseased parts of nail and soft tissue to be removed. It is simple, cost-effective, satisfactory and aesthetic. SBU/23.01.2019/B.10.1.TKH.4.34.H.GP.0.01/7 (retrospectively registered).

## Introduction

Nails have both functional and aesthetic importance. Their disorders are diagnosed mostly by clinical findings and they are very common in the daily practice of all the physicians who make outpatient clinics. Therefore, it is very important to be familiar with this entity. The most frequent nail disorders are ingrown toenail, nail fungus and trauma [1]. Local physical examination is usually enough to make a definitive diagnosis, but systemic diseases, such as diabetes mellitus (DM), peripheral vascular diseases or cardiac insufficiency, should be kept in mind. Unnoticed distal phalangeal fractures can also aggrevate the problem.

Narrow or improperly fitting shoes, incorrect nail-trimming, working at a job requiring long-time stand-up, obesity, genetical tendency, etc. may all result in ingrown toenail [2–4]. Change in the proper fit of the nail plate in the nail socket or irritation of the surrounding soft tissues by a spike of the nail usually causes a new granulation tissue and a feeling of chronic disturbing sensation. Since the nail will continue to grow, signs of inflammation (rubor, calor, tumor, dolor) become inevitable and increase day by day. Many methods from conservative approaches to radical surgical techniques have been described in the literature to overcome this frequently seen entity. Close observation, packing, taping, gutter treatment, nail braces, cauterization of the matrix or chemical matricectomies, partial or total nail extractions are among the treatment methods [5–10]. However, these treatment modalities have some disadventages, such as unnecessarily extensive surgery, need for chemical substanses that are not always available, etc. and side effects, such as excessive bleeding and infection. Furthermore, under- and over-treatments should be avoided, since both situations result in loss of labor and increase the treatment costs.

In the present study, we are going to present our experience gained from 2334 procedures in 2118 patients who were treated in outpatient clinics with a cost-effective, simple and practical easy to perform method with no special equipement needed.

## Patients and methods

The study was approved by our institutions’s Ethics’ Committe (SBU/23.01.2019/B.10.1.TKH.4.34.H.GP.0.01/7). All patients with ingrown toe who applied to outpatient clinics of General Surgery Department between 2013 and 2019 were enrolled, retrospectively. Data including demographics, uni- or bilaterality of lesions, history of local trauma and other predisposing factors together with comorbidities were documented through a detailed computer-based HIS (Health Information System) search. Informed consent was signed by each patient before surgical intervention.

Since our outpatient protocol dictates a multi-disciplinary approach in complicated cases with systemic comorbidities, such as diabetic foot, peripheral vascular disease, cardiac insufficiency, etc., these patients were excluded. Besides, patients who underwent total nail extractions due to fungus or trauma, and patients who do not come to control were also excluded from the study. However, ingrown toes due to local trauma were included.

**Fig. 1:**
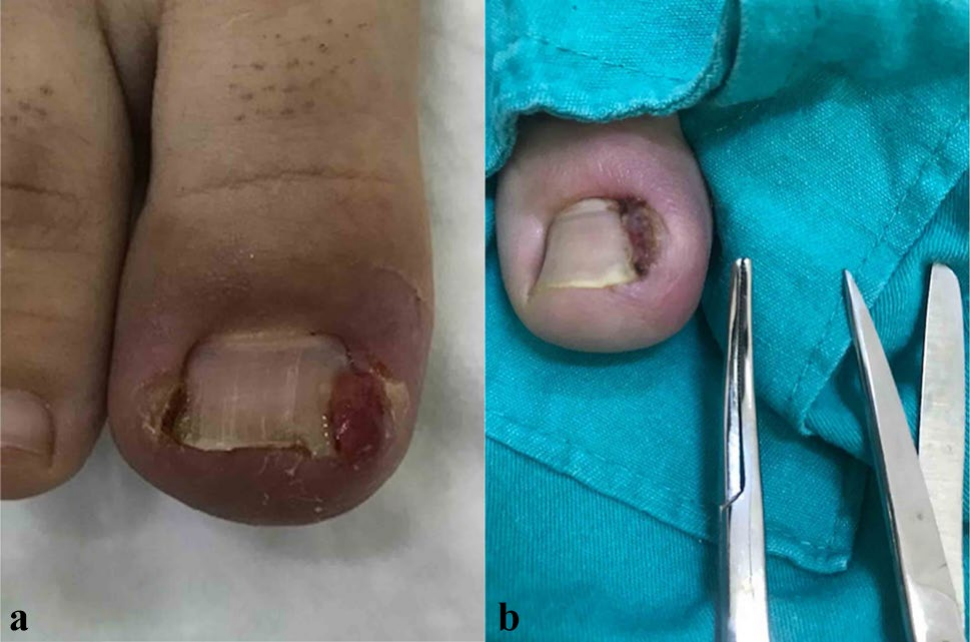
**a**, **b** Granulation tissue over the ingrown toe (local anesthesia, curved mosquito and tissue scissors)

**Fig. 2:**
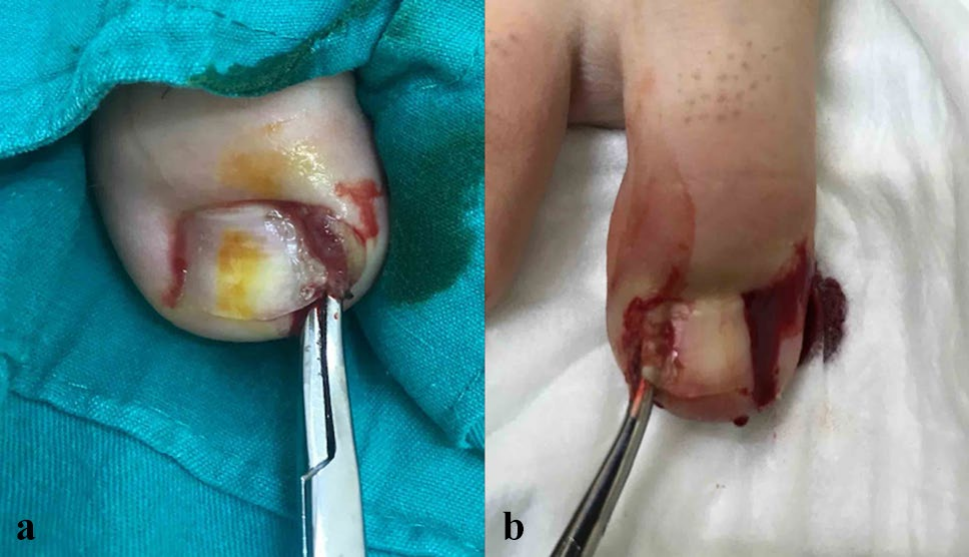
**a**, **b** Elevation of diseased nail side by inserting curved mosquito up to the root, touching the germinative matrix

**Fig. 3:**
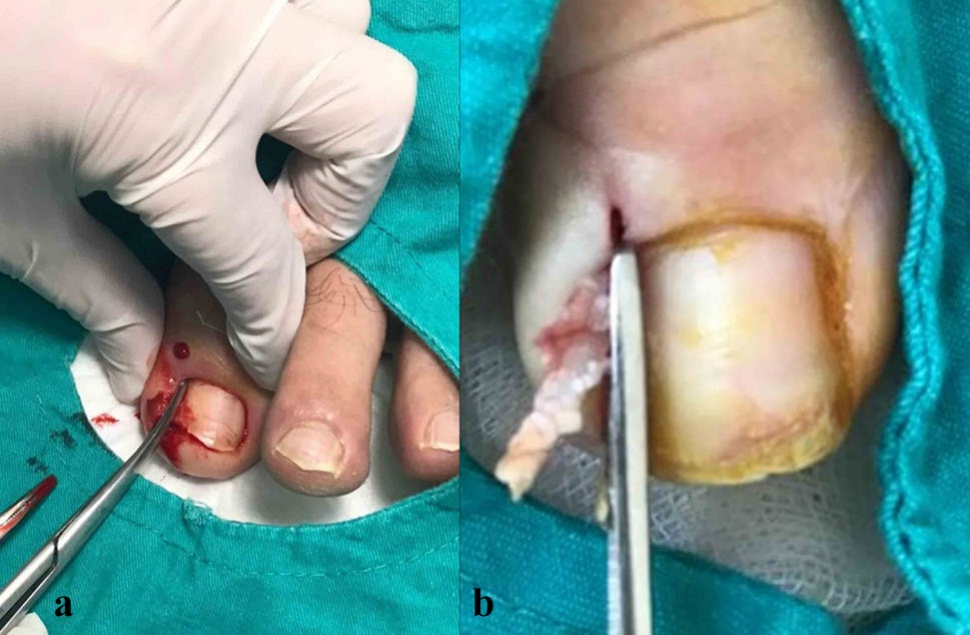
**a**, **b** Nail was cut straight lengthwise to the root (longitudinally) by a strong tissue scissors, taking whole diseased portion including the spikes with a minimal matricectomy

**Fig. 4:**
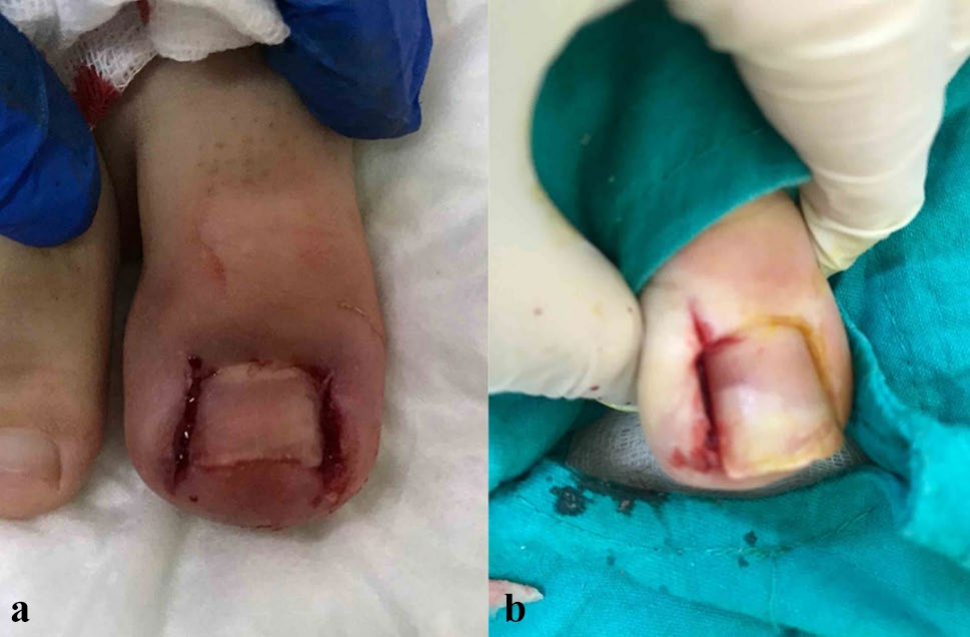
**a**, **b** No chemicals or electro-cautery was used to ablate the matrix, and a 2 mm-space was created between the healthy soft tissue and nail

**Fig. 5:**
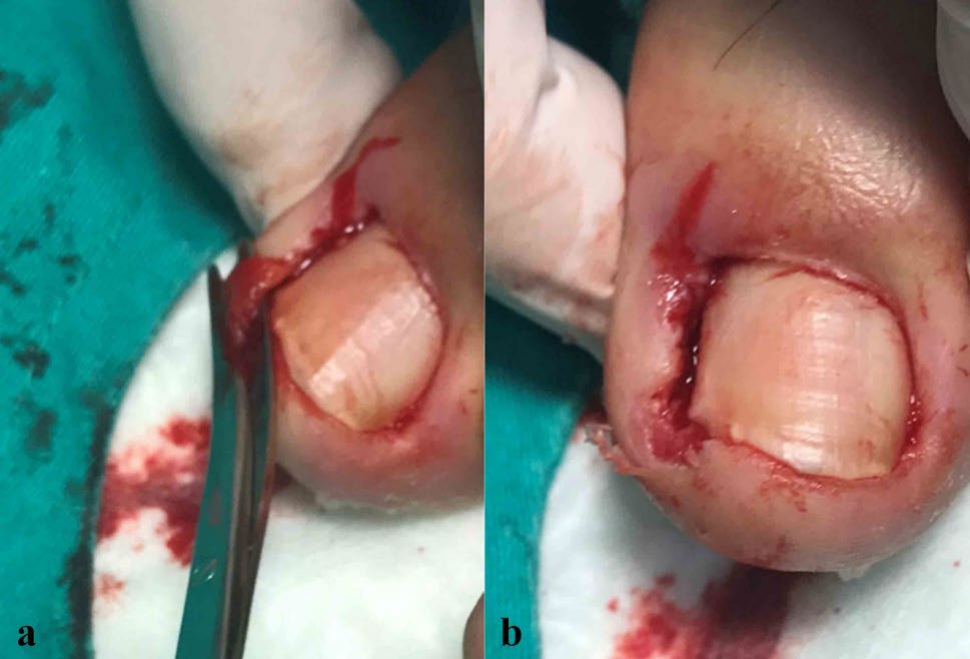
**a**, **b** In cases whom the adjacent soft tissue invades nail bed preventing the nail from growing, these excessive tissues were also shaved with scissors

All surgical procedures were performed by the same surgeon in outpatient conditions. We did not use tourniquet. Following 10% povidone iodine wipe and local anaesthesia (prilocaine); at first, the granulation tissue (if present) was debrided to see the anatomical position of nail and surrounding soft tissues (Fig. 1a, b). Then, a curved mosquito was used to elevate the nail plate by just inserting it beneath the ingrown-portion of nail up to the matrix (Fig. 2a, b). Nail was cut straight lengthwise to the root (longitudinally) by a strong tissue scissors, taking whole diseased portion including the spikes with a minimal matricectomy (Fig. 3a, b). The strong straight scissors ensured a smooth nail edge without any spicules. No chemicals or electro-cautery was used to ablate the matrix, and a 2 mm-space was created between the healthy soft tissue and nail (Fig. 4a, b). No suture was used. In cases whom the adjacent soft tissue invades nail bed preventing the nail from growing, these excessive tissues were also shaved with scissors (Fig. 5a, b). Then, the operative field was wrapped tightly for haemostasis, and we did not use any instrument, such as electro-cautery. The patients were adviced to come control or to change for a looser dressing on the following day.

All patients were prescribed aoral broad-spectrum antibiotic (ciprofloxacin) for prophylactic measures and an antibiotic-containing pomad (nitrofurazone), for 7 days, and a pain-killer. They were also adviced to keep their foot away from water for 7 days, and to put their foot in a bowl containing soapy warm water (for 20 min/day, during 1 week) after postoperative day 10.

Follow-up data were obtained from routine outpatient clinic controls at postoperative days 1, 10 and 30. The last follow-up was done by phone calls.

Statistical analysis was performed using SPSS Statistics 20 software (Windows 19th Version, Armonk, NY). Categorical variables were compared using Fisher’s exact test. Continuous variables were compared using Students’ *t* and Mann–Whitney *U* tests. *P* < 0.05 was considered statistically significant.

## Results

A total of 2334 surgical procedures were performed in 2118 patients. 54.2% of the patients was men and 45.7% was women (Table 1). Mean age was 27 ± 11 years for men and 26 ± 9.9 was for women. Recurrence rate was 1.7% (*n* = 41) during the mean follow-up period of 36 months (range 9–62). Most of the patients with recurrent ingrown toe were younger men (70.7%, mean 22 ± 4 years, *p* < 0.05 for each, Table 1). Most of the recurrences were seen in 3 months (range 1–5). The location of the lesions (right/left, medial/lateral or bilateral) did not show statistical difference (*p* > 0.05 for each). However, while the bilateral involvement constitutes only 4.5% of the total, this ratio increased to 14.6% in recurrent cases (*p* < 0.05).

**Table 1:** Demographics, location of lesions, recurrence rates

Demographics and location of lesions	Total number of ingrown toe (*n* = 2334)	Recurrence (*n* = 41, 1.7%)	*p*
Male/female	1149 (54.2%)/969 (45.7%)	29 (70.7%)*/12 (29.2%)	0.678
Age (median ± SD)	27 ± 11/26 ± 9.9	22 ± 4*/27 ± 7	0.411
Medial	1165 (49.9%)	23 (56%)	0.317
Lateral	1062 (45.5%)	12 (29.2%)	0.780
Bilateral (medial + lateral)	107 (4.5%)	6 (14.6)*	0.651
Side (right/left)	1190 (50.9%)/1144 (49%)	21 (51.2%)/20 (48.7%)	0.909

*p < 0.05 statistically significant

Predisposing factors for the recurrence were tight-fitting footwear (*n* = 96, 4.5%), incorrect nail-trimming (*n* = 65, 3%), genetic tendency (*n* = 61, 2.8%), obesity (body mass index-BMI > 30, *n* = 46, 2.1%) and trauma (*n* = 16, 0.75%), but each was statistically insignificant (*p* > 0.05).

Mean operation time, including local anesthesia, was 3 min (range 2–10). There was no important complication except hematoma/bleeding (*n* = 21, 0.89%) and infection/abscess (*n* = 16, 0.68%). All of these cases were managed well with a complete recovery. Mean healing time was estimated to be 10 days (7–21), and patients returned to their daily activities in 3 days (range 1–7).

## Discussion

Ingrown toenail, or onychocryptosis, is a very common problem and results from various etiologies, such as incorrect nail-trimming, poorly fitting shoes, poor foot hygiene, local trauma, genetical tendency, obesity, etc. [1, 2, 11]. It usually affects the big toe. Patients commonly present with pain in the affected nail, and signs of inflammation can be seen on physical examination. If definitive treatment is delayed, infection is inevitable. Most studies in the literature suggest a slightly higher male-to-female ratio, particularly in the 14–25 age group, but it can affect patients of any age [12]. In the present study, mean age was roughly around 27 years, but recurrence was mostly seen in younger males (mean 22 years). Most of the predisposing factors were positive, but there was no statistically significant relevance.

Diverse therapeutic options have been described in the literature. Conservative methods, such as packing, taping, gutter treatment and nail braces, are usually recommended for relatively mild cases, whereas surgery should only be done by physicians [4–10]. Wedge resections and partial or total extractions have been reported in some series, but recent publications favor mini-invasive surgery since most of the previous surgical techniques have caused over-treatment and do not guarantee that it will not relapse [13, 14]. In our series, we excluded the patients who underwent total extractions, because these patients had fungal infection or severe trauma causing whole nail in pieces or thickened. However, we prefered minimal invasive surgery even for bilateral ingrown nails, leaving a near-total portion of the nail intact.

Since ingrown toenail results from the compression of the medial and/or lateral nail folds on the nail plate, excision of the affected nail plate combined with proximal matricectomy is thought to provide the best chance for eradication. There is a general consensus that the remaining healthy nail plate should be preserved. However, the necessisity and extent of matricectomy is still debatable. Since we think that a successful nail surgery requires exposure of the underlying tissues, we debrided all dead/granulation tissues and removed the immersed nail spike(s) en block, up to the level of germinative matrix. We preferred minimal matricectomy together with unilateral longitudinal nail excision (en block), because remaining the matrix intact in place would not guarantee the total excision of the diseased, weaken and possibly spicule-containing proximal part of the nail. This would be an under-treatment with higher recurrence rates. In an other saying, the root of the nail, the closest part to the matrix layer, can be the main cause of ingrown toe. Infection or abscess is very common in this part of the finger, as well.

On the other hand, there are many methods of matricectomy described in the literature, e.g. electro-cautery ablation has been used to destroy the exposed nail-forming matrix, creating a new lateral nail fold [4, 15]. However, complications of the procedure include regrowth of a nail spicule secondary to incomplete matricectomy and postoperative nail bed infection, even under prophylactic antibiotherapy. A surgical instrument with electrode plate should also be available and be ready to use, as well. In our opinion, after excision of the problematic nail or matrix, it is not easy to see the exact point to be cauterized, due to bleeding. Cauterization does not guarantee a definitive cure since the matrixstay in its place, and the cauterized dead portion of matrix creates a focus of new infection, as well. Chemical matricectomy with phenol was also very popular for a while in the literature, as it has good cosmetic results [8]. However, it produces extensive tissue destruction that can result in serous or purulent drainage and delayed healing. Furthermore, phenol is not available in every clinic since it has been reported to be potentially cancerogenious [9]. Recently, Terzi et al. [16] recommended 90% trichloroacetic acid (TCA) in their 39 patients with 56 ingrown toenail edges after partial nail avulsion. They reported minimal complication rates. Similarly, Tatlıcan et al. [17] treated the germinal matrix with 10% sodium hydroxide for one minute, and reported good results. However, in our opinion, both reports have very limited patient numbers to make a conclusion. Furthermore, in both studies, the authors recommend partial avulsion surgery before chemical matricectomy. At this stage of operation, haemostasis has a pivotal importance, and it is not easy to touch matrix in a deeper anatomical location.

Similarly, in a study of Yabe et al. [18], operative microscope was used in 77 patients, and the authors have recommended minimal invasive methods to remove the germinal matrix. However, they did not answer how one might excise the already ingrown nail and formed granulation tissue. Some other recent studies with radiofrequency (RF) and carbon dioxide laser ablation of the nail matrix are also preliminary, and expensive options [19, 20]. Instead, en block resection of granulation tissue and diseased nail, with a mini-matricectomy, as shown in our series (over 2000 patients), is ideal for fulfilling all goals. It also supplies the best aesthetic outcome.

In conclusion, lateral longitudinal excision with minimal matricectomy technique guarantees all dead tissue and diseased parts (including nail and neighbouring soft tissue) to be removed and saves time for both physicians and patients. It is also simple, practical, cost-effective, aesthetic and satisfactory. However, the limitations of the present study were its retrospective design, the routine antibiotic prophylactic measures and the lack of in-person long-term follow-up.

## Data Availability

Currently forbidden by Ministry of Health to share, but in case of acceptance or reviewers want to see, we can send the excel by shading protocols and patient names.

## Code availability

L60.
